# Immune-Guided Bone Healing: The Role of Osteoimmunity in Tissue Engineering Approaches

**DOI:** 10.3390/ijms262311642

**Published:** 2025-12-01

**Authors:** Serena Munaò, Alessandra Armeli, Desirèe Bonfiglio, Antonella Iaconis, Giovanna Calabrese

**Affiliations:** Department of Chemical, Biological, Pharmaceutical and Environmental Sciences, University of Messina, Viale Ferdinando Stagno d’Alcontres, 31, 98168 Messina, Italy; serena.munao@studenti.unime.it (S.M.); alessandra.armeli@studenti.unime.it (A.A.); desiree.bonfiglio@studenti.unime.it (D.B.); iaconis.antonella@gmail.com (A.I.)

**Keywords:** osteoimmunology, bone immune system, bone tissue engineering, biomimetic scaffolds, immunoregulatory biomaterials

## Abstract

The skeletal and immune systems are intricately linked, forming a dynamic interface that regulates both bone homeostasis and immune function. This bidirectional relationship, central to the field of osteoimmunology, highlights how bone and immune cells interact via shared progenitors and signaling pathways. Osteoclasts and osteoblasts not only coordinate bone remodeling but also influence hematopoietic and immune functions within the bone marrow microenvironment. The concept of the “bone immune system” underscores this crosstalk, particularly in pathological and regenerative contexts. Despite progress, contradictory findings complicate our understanding of cytokine activity. Pro-inflammatory mediators such as TNF-α and IL-17 are typically associated with bone loss, yet under certain conditions, they paradoxically promote repair by stimulating osteoblast differentiation. Conversely, anti-inflammatory cytokines like IL-10 and TGF-β are generally protective, but their effects vary depending on local context, sometimes even impairing regeneration. These inconsistencies highlight unresolved questions and gaps in mechanistic insight into immune–bone interactions. Bone tissue engineering (BTE) has advanced through biomimetic scaffolds, osteogenic cells, and bioactive molecules, offering hope for large defect repair. However, clinical translation remains limited, largely because immune modulation is not fully integrated into scaffold design. Current preclinical models often fail to capture the complexity of immune–skeletal interplay, reducing predictive value. Addressing these gaps requires improved models and systematic evaluation of immunoregulatory biomaterials, paving the way for more effective and personalized regenerative therapies.

## 1. Introduction

The skeletal and immune systems are finely interconnected, and their reciprocal regulation forms the basis of osteoimmunology, a discipline that investigates how immune mechanisms influence bone physiology and pathology [1,2].

Bone is not only a structural organ but also a dynamic tissue that supports mineral metabolism, hematopoiesis, and endocrine signaling, thereby linking skeletal integrity to systemic homeostasis [3,4,5].

Recent advances have revealed that immune-derived signals critically affect bone remodeling, influencing osteoclast and osteoblast activity and determining outcomes in both health and disease [6,7,8,9,10,11]. This interplay has great implications for regenerative medicine, particularly in the context of bone tissue engineering (BTE), where immune responses dictate the integration and functionality of biomaterials [12,13,14,15,16,17].

The aim of this review is to provide a comprehensive overview of bone–immune interactions, with a particular focus on their role in remodeling and regeneration. We highlight the cellular and molecular mechanisms that govern osteoimmunity, examine how engineered bone constructs modulate local immune responses, and discuss the importance of designing biomaterials with immunoregulatory properties. Finally, we emphasize the need for improved preclinical models capable of assessing immunological outcomes, which will be essential for translating osteoimmunology-informed strategies into effective clinical therapies.

## 2. Bone Remodeling and Osteoimmunity

### 2.1. Overview of Bone Remodeling

Bone is a mineralized connective tissue composed of a collagen-rich matrix, vascular and neural networks, and four primary bone cell types: osteoblasts, osteocytes, osteoclasts, and osteogenic progenitors [18,19]. These cells coordinate the dynamic process of bone remodeling through osteoclast-mediated resorption and osteoblast-driven formation. Beyond structural support, bone performs multiple physiological roles, including organ protection, locomotion, mineral storage (particularly calcium and phosphorus), and blood cell production within the bone marrow niche [20,21].

This multifunctionality highlights its integration with other physiological systems, particularly the immune system.

### 2.2. Bone–Immune Interactions

Bone and immune systems share developmental origins and signaling pathways [22]. Osteoclasts, the bone-resorbing cells, originate from myeloid precursors common to macrophages and dendritic cells, while osteoblasts, responsible for bone formation, regulate hematopoietic stem cell niches. Within the bone marrow microenvironment, bone, immune, and stromal cells communicate through cytokines, chemokines, and growth factors that modulate remodeling [23].

**Figure 1:**
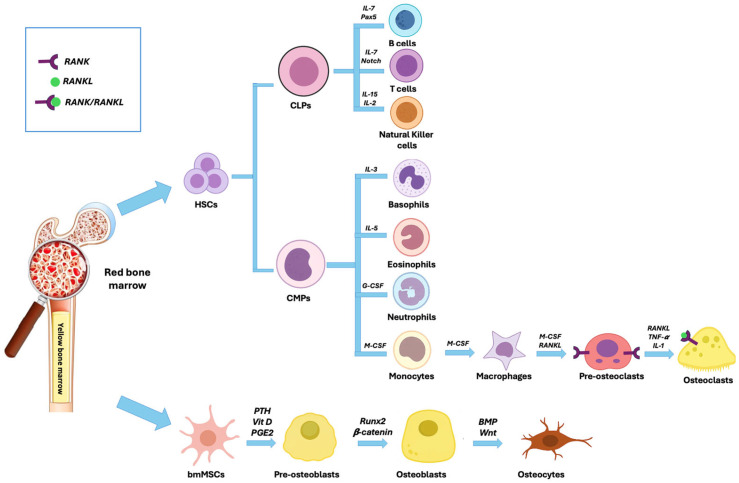
Lineage commitment and differentiation of hematopoietic stem cells (HSCs) and bone marrow mesenchymal stem cells (bmMSCs) within the red bone marrow microenvironment. HSCs give rise to common lymphoid progenitors (CLPs) and common myeloid progenitors (CMPs). CLPs differentiate into: B cells, T cells, and natural killer (NK) cells. CMPs generate basophils, eosinophils, neutrophils and monocytes. Monocytes further differentiate into macrophages and finally into osteoclasts. Concurrently, bmMSCs differentiate into pre-osteoblasts, which mature into osteoblasts, and subsequently into osteocytes.

This integrated network, known as “bone immune system” links skeletal integrity with immune function, a paradigm defined as osteoimmunity (Figure 1) [24,25,26].

### 2.3. Bone Composition and Cellular Organization

The skeleton undergoes lifelong remodeling to maintain bone strength and mineral homeostasis. This physiological process is governed by a dynamic equilibrium of bone resorption and formation, orchestrated by complex interactions among various cell types [20].

The extracellular matrix of bone consists of type I collagen (organic component), providing tensile strength and structural integrity, and hydroxyapatite crystals (inorganic portion), conferring rigidity and compressive strength [27].

The principal cellular components of bone include osteoblasts, which synthesize and deposit matrix osteoclasts, which are specialized in bone resorption, and osteocytes, embedded within the matrix as mechanosensors that regulate bone turnover [21,28]. Osteogenic progenitors further contribute by differentiating into osteoblasts and contributing to bone regeneration [29]. Additional contributors, such as stromal, cartilage, hematopoietic, and mesenchymal stem cells, participate in bone composition, forming a dense signaling network [30,31].

Bone remodeling occurs within specialized anatomical structures known as basic multicellular units (BMUs), where bone-lining cells create microenvironments for sequential resorption and formation [32,33,34].

**Figure 2:**
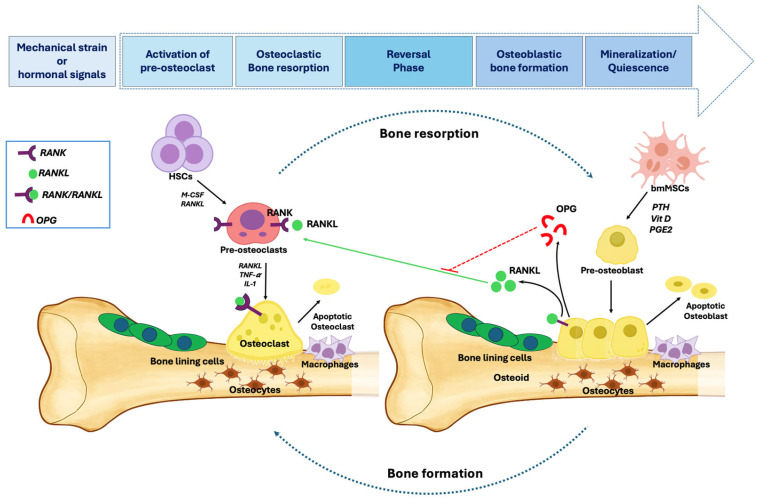
Cellular and Molecular Mechanisms of Bone Remodeling. Bone remodeling occurs sequentially at the same site, with osteoclast-mediated resorption followed by osteoblast-driven bone formation. Hematopoietic stem cells (HSCs) give rise to pre-osteoclasts under M-CSF and RANKL, maturing into osteoclasts that resorb bone. Bone marrow mesenchymal stem cells (bmMSCs) differentiate into pre-osteoblasts under PTH, VD3, and PGE2, maturing into osteoblasts that produce osteoid and become osteocytes. Macrophages clear apoptotic cells during the reversal phase. Osteoblast-derived osteoprotegerin (OPG) inhibits RANKL-RANK signaling, regulating osteoclastogenesis.

The cycle begins with activation, triggered by mechanical strain or hormonal signals such as estrogen or parathyroid hormone, which are detected by osteocytes [35,36] (Figure 2). This initiates resorption, driven by osteoclast precursors. Following osteoclast apoptosis, the reversal phase begins, with osteoblasts synthesizing new matrix to fill the cavities left by resorption. The final mineralization phase calcifies the matrix, after which osteoblasts undergo apoptosis, become bone-lining cells, or are incorporated into the matrix as osteocytes, entering quiescence [37,38].

Immune-derived signals strongly influence these phases [39,40]. Pro-inflammatory cytokines such as interleukin-1 (IL-1), interleukin-6 (IL-6), and tumor necrosis factor-alpha (TNF-α) promote osteoclastogenesis, thereby enhancing bone resorption. In contrast, anti-inflammatory mediators, including interferon-beta (IFN-β), interferon-gamma (IFN-γ), interleukin-4 (IL-4), interleukin-12 (IL-12), and interleukin-18 (IL-18), suppress osteoclast formation and activity, supporting bone preservation and maintaining remodeling balance [41,42,43].

### 2.4. Bone Tissue Engineering and Regenerative Implications

Although bone can naturally regenerate, exceed this capacity, creating a major clinical challenge for millions of patients [44,45]. BTE addresses this by combining biocompatible scaffolds, osteogenic cells, and bioactive molecules to promote functional bone regeneration [46]. Recent advances, including nanotechnology-enhanced scaffolds and 3D-printed biomaterials, have demonstrated improved osteointegration and cellular responses [47,48]. However, the success of BTE depends not only on the material and cellular components but also on immune modulation. Immune cells play a pivotal role in determining the fate of implanted constructs, influencing both their integration and the quality of newly formed bone [49]. Understanding bone–immune crosstalk is therefore essential for advancing regenerative therapies and developing immuno-informed biomaterials.

## 3. The Immune System and Its Role in Bone Biology

### 3.1. Immune Cell Types and Signaling Pathways

The immune system is a complex network of cells, tissues, and signaling molecules that defends the body against pathogens and maintains tissue homeostasis [50]. Within the bone marrow, hematopoietic stem cells (HSCs) differentiate into a broad spectrum of immune cells, including macrophages, dendritic cells, neutrophils, T cells, B cells, and natural killer (NK) cells. These immune cells not only contribute to host defense but also play pivotal roles in bone remodeling and regeneration by interacting with bone-resident cells and influencing their behavior [51,52].

Pattern recognition receptors (PRRs), such as Toll-like receptors (TLRs), NOD-like receptors (NLRs), and RIG-I-like receptors (RLRs), detect pathogen-associated molecular patterns (PAMPs) and danger-associated molecular patterns (DAMPs) within the bone microenvironment. Their expression on both immune and bone cells allows direct modulation of remodeling [53].

### 3.2. Regulation of Osteoclastogenesis: Systemic and Local Signals

Osteoclastogenesis in vivo is regulated by a hierarchical network of systemic hormones and local cytokines that converge on the RANK/RANKL/OPG signaling axis to control osteoclast precursor differentiation, activity, and survival [54].

Systemic signals act through endocrine mechanisms and include parathyroid hormone (PTH), 1α,25-dihydroxyvitamin D_3_, and soluble RANKL. These factors stimulate bone cells to produce local mediators, thereby amplifying osteoclastogenic responses within the bone microenvironment. For example, PTH directly regulates RANKL gene expression in osteocytes [55], while 1α,25-dihydroxyvitamin D_3_ modulates RANKL and OPG expression in synoviocytes and osteoblasts [56]. Soluble RANKL itself circulates and binds to RANK on osteoclast precursors, initiating differentiation.

Local signals are produced by bone-resident and immune cells and act in a paracrine or autocrine manner. Membrane-bound RANKL expressed by osteoblasts, osteocytes, and activated T cells is a central mediator of osteoclastogenesis. Pro-resorptive cytokines such as TNF-α, IL-1, IL-6, IL-11, IL-17, and IL-7 promote osteoclast differentiation by upregulating RANKL and suppressing OPG. TNF-α, for instance, directly enhances RANKL expression and inhibits OPG production in osteocytes [57,58], while IL-1 and IL-6 activate NF-κB signaling and stimulate RANKL production [59,60,61]. IL-11, produced by stromal cells, supports osteoclastogenesis by increasing RANKL expression and facilitating bone resorption [62,63]. IL-17, secreted by Th17 cells, mediates PTH-induced bone loss through IL-17 receptor signaling [64,65], and IL-7 indirectly increases RANKL expression by activating the c-Fos/c-Jun pathway in T cells [41,66]. Prostaglandin E_2_ also contributes by influencing osteoblast and osteoclast activity through EP4 receptor signaling [67,68]. In addition, macrophage colony-stimulating factor (M-CSF) is essential for osteoclast precursor survival and upregulates RANK expression, working synergistically with RANKL to drive differentiation [69,70].

In contrast, anti-resorptive cytokines (IL-13, IFN-γ, IL-4, IL-10, IL-18, IL-33, TGF-β) act as inhibitors of osteoclast formation and activity. IL-13 and IL-4 stimulate OPG production via the STAT6 pathway, counteracting RANKL-mediated osteoclastogenesis [41,71]. IL-4 suppresses osteoclast formation both in vitro and in vivo [72]. IFN-γ interferes with RANKL signaling by degrading TRAF6 and suppressing RANK and CSF1R expression in osteoclast precursors [73,74]. IL-10 modulates both osteoclast and osteoblast activity, altering the RANKL/OPG ratio and suppressing mineralization [75]. IL-18 indirectly inhibits osteoclast differentiation by stimulating GM-CSF production from naïve T cells [76,77]. IL-33 exhibits dual effects, functioning as an anti-osteoclastogenic factor in some contexts while synergizing with IL-6 to promote bone resorption in others [78]. Transforming growth factor-beta (TGF-β), produced by macrophages and other cell types, reduces RANKL, enhances OPG and modulates the release of pro-inflammatory cytokines, thereby promoting bone stability [79].

Table 1 provides an overview of the key systemic and local cytokines and factors involved in osteoclastogenesis and bone resorption. It highlights their specific regulatory effects on the RANK/RANKL/OPG signaling axis, identifies their primary cellular sources, and outlines their impact on bone remodeling.

Downstream of these systemic and local signals, RANKL binding to RANK activates intracellular pathways including NF-κB, MAPK, and AP-1 (c-Fos/c-Jun), which drive the transcription of genes essential for osteoclast differentiation and function. NFATc1, the master transcription factor of osteoclastogenesis, integrates these signals to orchestrate the formation and activity of mature osteoclasts [70,80].

**Figure 3:**
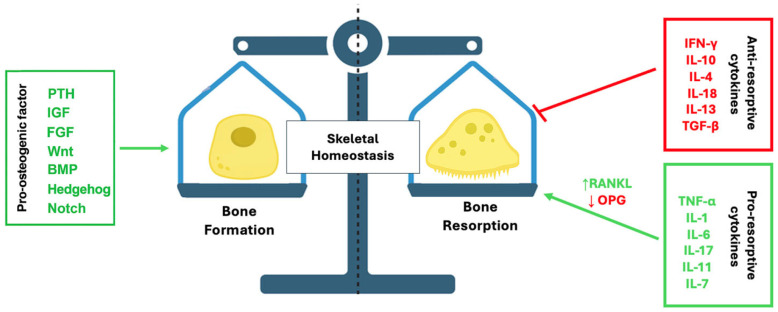
Molecular regulation of skeletal homeostasis. The image illustrates the balance of factors and cytokines that regulate skeletal homeostasis by influencing bone formation and bone resorption. Bone formation is stimulated by pro-osteogenic factors such as PTH, IGF, FGF, Wnt, BMP, Hedgehog, and Notch. Bone resorption is promoted by pro-resorptive cytokines like TNF-α, IL-1β, IL-6, IL-7, and IL-17. Anti-resorptive cytokines including IFN-γ, IL-4, IL-10, IL-12, IL-18, IL-33, TGF-β inhibit osteoclastogenesis.

This integrated framework highlights how systemic factors initiate osteoclastogenic signaling, local mediators amplify and fine-tune the response, and intracellular pathways execute the differentiation program, ensuring that bone resorption remains tightly regulated within skeletal homeostasis (Figure 3).

### 3.3. The RANK/RANKL/OPG Axis

The RANK/RANKL/OPG pathway is central to bone remodeling and osteoimmunology. RANKL, expressed by osteoblasts, stromal cells, and activated T cells, binds to its receptor RANK on osteoclast precursors, triggering a cascade of intracellular events that promote osteoclast differentiation and resorption. Macrophage colony-stimulating factor (M-CSF) enhances this process by upregulating RANK and supporting precursor fusion into multinucleated osteoclasts capable of degrading bone matrix [81,82,83,84].

OPG, secreted by osteoblasts and B cells, acts as a decoy receptor binding RANKL with high affinity and preventing its interaction with RANK. This competitive inhibition suppresses osteoclastogenesis and protects bone from excessive degradation [82]. The RANKL/OPG balance determines skeletal homeostasis and is influenced by a variety of cytokines, hormones, and mechanical stimuli. Dysregulation contributes to osteoporosis, rheumatoid arthritis, and metastatic bone disease, making this axis a major therapeutic target [85].

## 4. Osteoimmunology: Bridging Bone and Immunity

### 4.1. Shared Origins of Osteoclasts and Immune Cells

Osteoclasts, the specialized bone-resorbing cells, originate from HSCs within the bone marrow, specifically the monocyte/macrophage lineage. This shared origin explains the immunological behavior, including responsiveness to cytokines and capacity to modulate immune responses [86].

T cells, particularly activated CD4+ and CD8+ subsets, can influence osteoclast differentiation through RANKL binding to RANK on osteoclast precursors [7], while activated T cells also regulate osteoclastogenesis via the RANK/RANKL/osteoprotegerin (OPG) axis [87].

Conversely, bone cells regulate immune function. Osteoblasts and osteocytes produce cytokines and growth factors such as IL-7 and CXCL12, which are essential for B cell maturation and T cell homing within the bone marrow [88].

Macrophages play a central role in osteoblast differentiation, partially through the secretion of oncostatin M [89]. Bone-resident macrophages, named osteomacs, have emerged as key regulators of osteoblast function and bone formation.

Recent studies show that osteoclasts are not merely skeletal cells but also possess immune-like functions. They can present antigens, influence T cell activation, and participate in inflammatory signaling [1,90]. This bidirectional communication is especially evident in pathological conditions [19]. In autoimmune diseases such as rheumatoid arthritis, aberrant immune activation leads to excessive osteoclast activity and bone erosion. Similarly, chronic inflammation alters bone remodeling, contributing to osteoporosis and impaired fracture healing [91,92]. This dual identity of osteoclasts reshapes our understanding of bone remodeling, emphasizing the deep integration of skeletal and immune systems in both health and disease.

### 4.2. Cytokines, Chemokines, and Growth Factors in Bone–Immune Crosstalk

The bone microenvironment is a dynamic niche where immune cells and bone cells communicate through cytokines, chemokines, and growth factors. This crosstalk is central to maintaining skeletal integrity and responding to physiological and pathological stimuli.

Anti-resorptive cytokines such as IL-4, IL-10, IL-13, IFN-γ, and IL-18 preserve bone mass by modulating the RANK/RANKL/OPG axis and inhibiting osteoclast activity.

Chemokines and growth factors also contribute to bone–immune crosstalk. CXCL12 and CCL19 regulate immune cell migration within the bone marrow, influencing hematopoiesis and immune surveillance. M-CSF and RANKL are critical for osteoclast differentiation and survival, while TGF-β and bone morphogenetic proteins (BMPs) promote osteoblast activity and bone formation [93].

B cells contribute significantly to bone homeostasis, producing ~64% of total OPG under physiological conditions. However, in aging and inflammatory states, B cell-derived RANKL and OPG levels may shift toward promoting bone loss [94].

## 5. Pathological Implications of Osteoimmune Dysregulation

Osteoimmune dysregulation refers to the disruption of the delicate balance between immune signaling and bone remodeling. Under physiological conditions, immune and bone cells participate in coordinated crosstalk that maintains skeletal integrity. However, chronic inflammation, autoimmunity, infection, or malignancy can disturb this communication, leading to pathological bone loss or abnormal bone formation.

### 5.1. Skeletal Diseases and Immune-Mediated Bone Loss

Osteoporosis is one of the most common bone disorders, characterized by reduced bone mass, compromised microarchitecture, and increased fracture risk [95,96]. While classical causes include calcium deficiency, endocrine dysfunction, and corticosteroid use, chronic inflammation has emerged as a key driver of bone loss [97]. Pro-inflammatory cytokines, particularly IL-1, IL-6, and TNF-α, enhance osteoclast activity and suppress osteoblast function, shifting the balance toward bone resorption. The term immunoporosis has been introduced to describe osteoporosis driven by immune dysregulation, underscoring the therapeutic potential of targeting cytokine pathways [98].

Rheumatoid arthritis (RA) provides a direct example of osteoimmune interaction. This autoimmune disease is marked by chronic joint inflammation, synovial hyperplasia, and progressive bone erosion. CD4+ T cells lose tolerance to modified self-antigens, triggering a cascade of cytokine release, including IL-1, IL-2, IL-6, IL-17, TNF, and IL-23, that amplifies inflammation and enhances RANKL expression. Th17 cells play a central role by secreting IL-17, which recruits macrophages and neutrophils, further promoting osteoclast activation and bone resorption [99].

Periodontal disease also exemplifies immune-driven bone loss. Activated B and T cells in gingival tissues produce RANKL, stimulating osteoclast-mediated degradation of alveolar bone [100].

### 5.2. Systemic Conditions Affecting Skeletal Homeostasis

Diseases not traditionally associated with the skeleton can still disrupt bone homeostasis due to the close interaction between immune and bone cells [1].

In cancer, the bone marrow microenvironment supports tumor growth and metastasis. Tumor cells, especially in breast and prostate cancers, secrete PTH-related peptide, which upregulates RANKL and promotes osteolysis. This process releases growth factors from bone that further fuel tumor progression. As a result, targeting the RANK/RANKL/OPG axis has become a promising therapeutic strategy in oncology [101].

In diabetes, the concept of mobiliopathy describes impaired mobilization of hematopoietic stem/progenitor cells (HSPCs). Diabetic patients often exhibit altered bone marrow niches that reduce vascular repair and bone regeneration. Dysfunctional bone marrow-derived mesenchymal stem cells (BMSCs), contribute to poor bone healing and remodeling [102].

Neurogenic heterotopic ossification (NHO) involves abnormal bone formation in soft tissues following central nervous system injury. Although the mechanisms remain unclear, immune modulation may offer therapeutic potential in preventing or treating NHO [103].

HIV infection and immunosuppressive therapy also impact skeletal health. HIV severely compromises immune function and is associated with bone deterioration. Antiretroviral therapy can paradoxically induce bone loss by reactivating immune responses that stimulate osteoclastogenesis. Inflammatory rebound during immune reconstitution may further exacerbate bone resorption, illustrating the complex interplay between immune restoration and skeletal integrity [104].

## 6. Immunomodulation in Bone Regeneration

Bone regeneration is a complex process that depends not only on osteogenic cells and biomaterials but also on the orchestration of immune responses [105]. The emerging field of osteoimmunomodulation highlights the critical role of immune cells and their signaling molecules play a pivotal role in scaffold integration, tissue repair, and long-term healing. Rather than suppressing immune activity, modern strategies aim to guide and harness it to promote regeneration [106].

### 6.1. Bone Scaffolds for Immunomodulation

**Figure 4:**
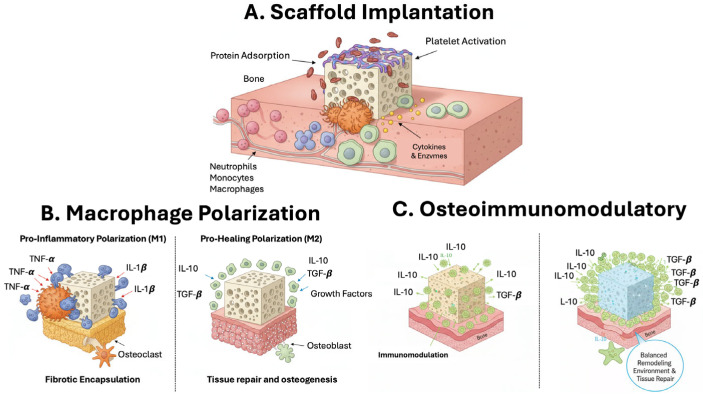
Immunomodulatory Interactions Between Bone Scaffolds and the Host Immune System.

Biomaterial implants and medical devices are widely used to repair or regenerate damaged tissues. In bone tissue engineering, scaffolds serve as substrates to deliver cells, provide biological signals, and activate endogenous repair mechanisms [107]. Ideal scaffolds must be biocompatible, non-toxic, and non-carcinogenic, with strong osteoconductive and osteoinductive properties. Common materials include natural polymers (e.g., collagen, chitosan), synthetic polymers (e.g., polyesters), ceramics (e.g., bioglass, calcium phosphate), metals (e.g., titanium alloys), and composites [108]. Upon implantation, biomaterials interact with the host immune system. The process triggers an inflammatory response, inducing protein adsorption and platelet activation, followed by recruitment of innate immune cells, neutrophils, monocytes, and macrophages. These cells secrete cytokines and enzymes that influence scaffold integration [109] (Figure 4).

Macrophages play a dual role. They help clear debris and orchestrate repair, but can also drive chronic inflammation and fibrotic encapsulation, known as the foreign body response. This response may isolate the implant in a dense extracellular matrix capsule, preventing osseointegration and rendering the scaffold nonfunctional [110].

To overcome this, new biomaterials are being designed with osteoimmunomodulatory (OIM) properties. These materials actively modulate the local immune environment to favor osteogenesis and improve scaffold integration [111,112].

### 6.2. Scaffold-Based Strategies for Local Immune Modulation

**Figure 5:**
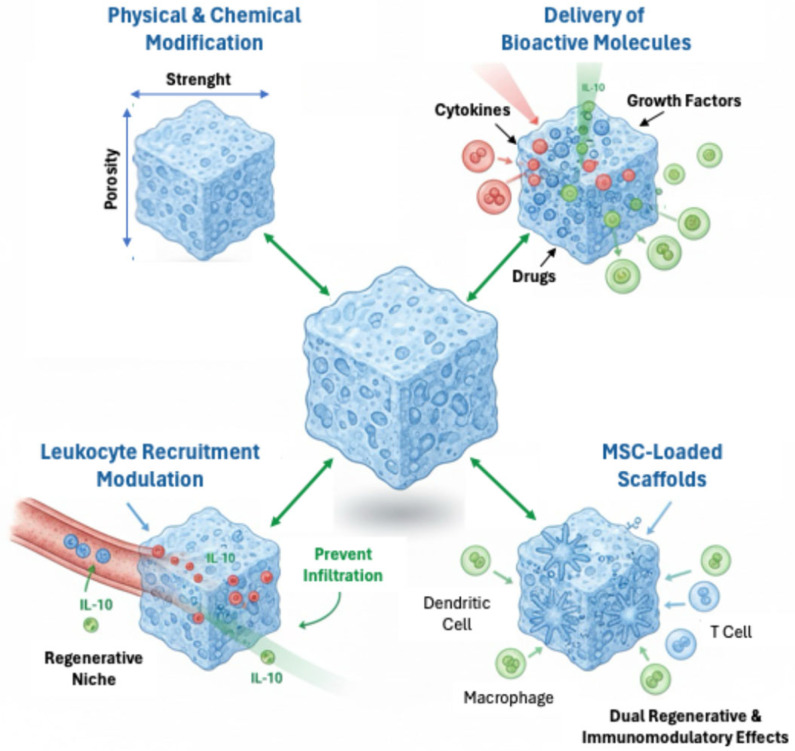
Scaffold-Based Strategies for Local Immune Modulation. Schematic representation of key scaffold-based strategies used to modulate the local immune microenvironment and enhance bone regeneration.

To enhance bone regeneration, a variety of scaffold-based strategies have been developed to actively modulate the local immune microenvironment (Figure 5).

These approaches reflect the shift toward immunologically informed biomaterials that engage the immune system to optimize healing.

One primary approach involves the physical and chemical modification of scaffold properties. Parameters such as stiffness, geometry, hydrophilicity, surface charge, pore size, and porosity have been shown to significantly influence immune cell behavior and cytokine secretion, thereby shaping the inflammatory response and promoting tissue integration [109,113]. For example, surface topography and mechanical cues can guide macrophage polarization and dendritic cell activation, contributing to a pro-regenerative immune niche.

Another key approach involves engineering scaffolds to facilitate the controlled release of therapeutic agents, such as proteins, cytokines, drugs, and peptides, that suppress inflammation and direct immune cell behavior. Anti-inflammatory interleukins such as IL-4 and IL-10 promote M2 macrophage polarization, which supports tissue repair and limits recruitment of pro-inflammatory cells. Co-delivery of glucocorticoids with IL-6 or IL-10 has demonstrated efficacy in reducing peri-implant inflammation and improving scaffold integration [112].

Modulating leukocyte recruitment is another promising possibility. Cytokines like IL-10 can prevent infiltration of pro-inflammatory leukocytes, helping establish a regenerative immune microenvironment. This is especially beneficial in chronic inflammatory conditions, where excessive immune activation can impair scaffold performance [110].

A biologically sophisticated strategy involves the incorporation of MSCs into scaffolds. MSCs offer both regenerative and immunoregulatory benefits. They secrete anti-inflammatory cytokines such as TGF-β1 and IL-10, and modulate the behavior of macrophages, dendritic cells, and T cells. These interactions promote a pro-healing immune response. In vivo studies have shown that MSC-loaded scaffolds can mitigate foreign body responses, limit fibrosis, and enhance tissue repair [107].

### 6.3. Hydrogel Systems and Advanced Delivery Platforms

Hydrogels are emerging as versatile platforms for immunomodulation due to their intrinsic biocompatibility and highly tunable physicochemical properties. They can respond to environmental stimuli, such as changes in pH, temperature, or inflammatory signals, to release therapeutic agents precisely when and where needed.

Incorporating nanoparticles into hydrogels enhances structural stability and allows sequential or sustained delivery of bioactive molecules [114,115]. One of their key advantages is the ability to restore immune homeostasis at injury site. Hydrogels modulate the local immune microenvironment, balancing effector and regulatory immune cell activity to promote tissue repair and prevent chronic inflammation [19]. This immunoregulatory capacity, combined with spatially targeted release, makes hydrogels ideal for localized bone regeneration. They improve therapeutic precision and reduce systemic side effects, positioning them as a cornerstone of next-generation regenerative biomaterials [115].

## 7. Current Challenges and Future Perspectives

Despite the promising advances in biomaterial design and hydrogel-based immunomodulatory platforms, several challenges remain before these technologies can be fully translated into clinical practice. One of the primary barriers is the complexity of the immune response in human patients, which varies significantly across individuals and disease conditions. Translating preclinical success into clinical outcomes requires rigorous validation, scalable manufacturing processes, and alignment with regulatory standards [49,116].

Personalized approaches in osteoimmunomodulation are gaining attention. These strategies aim to tailor biomaterial properties and therapeutic payloads to match each patient’s immune profile and bone healing needs. Precision-based strategies hold promise for treating complex conditions such as non-union fractures, autoimmune-related bone loss, and aging-associated regeneration deficits [106,112].

Looking ahead, emerging technologies, including bioresponsive hydrogels, 3D bioprinting, and AI-guided design, are set to revolutionize the field. These innovations, coupled with interdisciplinary collaborations among immunologists, materials scientists, and clinicians, will be essential to unlock the full therapeutic potential of immunomodulatory biomaterials in bone regeneration [117].

## 8. Conclusions

Osteoimmunity has emerged as a pivotal regulator in bone tissue engineering (BTE), reshaping our understanding of how immune responses influence skeletal repair. The intricate interplay between immune and bone-forming cells influences inflammation, healing, biomaterial integration and long-term regeneration. This evolving knowledge has led to the development of immunomodulatory strategies that harness the immune system as a therapeutic partner rather than a barrier.

Osteoimmunology also offers a framework for identifying specific molecular and cellular targets in the treatment of bone diseases. By decoding immune signaling pathways, researchers can modulate their impact on bone remodeling and repair. This deeper understanding of immune–bone crosstalk enables the design of innovative therapeutic approaches, particularly in the context of scaffold-based interventions.

Looking forward, immune-guided bone healing strategies offer a transformative approach to regenerative medicine. Biomaterials such as hydrogels, bioactive scaffolds, and smart delivery systems are being engineered to interact with and modulate the immune microenvironment. The incorporation of molecules like anti-inflammatory cytokines or immunoregulatory peptides enhances the regenerative potential of next-generation bone implants.

As technologies evolve and interdisciplinary collaborations deepen, osteomodulatory biomaterials are poised to become important tools in advanced BTE. These innovations bridge biological complexity and clinical precision, offering personalized, efficient, and targeted solutions for skeletal regeneration.

## Figures and Tables

**Table 1:** Systemic and local factors regulating osteoclastogenesis through the RANK/RANKL/OPG axis.

Cytokines	Factor Effect on RANK/RANKL/OPG Axis	Immune Cell Source	Impact on Bone	Ref.
**Parathyroid** **Hormone** **(PTH)**	↑ RANKL	Parathyroid gland (chief cells)	Stimulates osteoclastogenesis	[55]
**1α,25-dihydroxyvitamin** **D_3_**	↑ RANKL, ↓ OPG	Synoviocytes	Enhances osteoclast differentiation	[56]
**TNF-α**	↑ RANKL, ↓ OPG	Macrophages, T cells	Potent enhancer of bone resorption	[57,58]
**IL-1**	↑ RANKL, ↓ OPG	Macrophages, Monocytes	Drives inflammatory bone loss	[42,60]
**IL-6**	↑ RANKL	T cells, Macrophages	Promotes osteoclast activity	[41,61]
**IL-11**	↑ RANKL	Stromal cells	Supports osteoclastogenesis	[62,63]
**IL-17**	↑ RANKL	Th17 cells	Strongly pro-resorptive	[64,65]
**IL-7**	↑ RANKL (indirectly)	T cells	Contributes to osteoclast precursor priming	[41,66]
**Prostaglandin E_2_** **(PGE_2_)**	↑ RANKL	Macrophages, stromal cells	Promotes bone resorption	[67,68]
**M-CSF**	↑ RANKL expression	Macrophages, stromal cells	Facilitates osteoclast precursor survival	[69,70]
**IL-13**	↓ RANKL, ↑ OPG	Th2 cells	Suppresses osteoclast formation	[41,71]
**IL-4**	↓ RANKL; inhibits osteoclast differentiation	Th2 cells	Potently suppresses osteoclast formation	[41,71,72]
**IFN-γ**	↓ RANKL signaling, inhibits stimulatory cytokines	T cells	Blocks osteoclast differentiation	[73,74]
**IL-10**	↓ NFATc1, alters RANKL/OPG ratio	Macrophages, T cells B cells	Inhibits osteoclastogenesis and mineralization	[75]
**IL-18**	↑ IFN-γ, ↑ GM-CSF	Dendritic cells, macrophages	Indirectly suppresses osteoclast precursors	[76,77]
**IL-33**	↑ IFN-γ, ↑ GM-CSF	Dendritic cells, macrophages	Indirectly suppresses osteoclast precursors	[78]
**TGF-β**	↓ RANKL, ↑ OPG; modulates inflammatory cytokines	Macrophages, stromal cells	Anti-resorptive, promotes bone stability	[79]

Symbols: ↑ = increase, ↓ = decrease.

## Data Availability

No new data were created or analyzed in this study. Data sharing is not applicable to this article.

## Abbreviations

The following abbreviations are used in this manuscript:

BTE	Bone Tissue Engineering
BMSCs	Bone marrow stromal cells
HSCs	Hematopoietic stem cells
BMUs	Basic multicellular units
IL-1	Interleukin-1
IL-6	Interleukin-6
TNF-α	Tumor necrosis factor-alpha
IFN-β	Interferon-beta
IFN-	Interferon-gamma
IL-4	Interleukin-4
IL-12	Interleukin-12
IL-18	Interleukin-18
NK	Natural killer
PRRs	Pattern recognition receptors
TLRs	Toll-like receptors
NLRs	NOD-like receptors
RLRs	RIG-I-like receptors
PAMPs	Pathogen-associated molecular patterns
DAMPs	Danger-associated molecular patterns
IL-17	Interleukin-17
IL-10	Interleukin-10
IL-13	Interleukin-13
PTHrP	Parathyroid hormone-related peptide
PGE2	Prostaglandin E2
OPG	Osteoprotegerin
RANKL	Receptor activator of nuclear factor κB ligand
M-CSF	Macrophage colony-stimulating factor
PTH	Parathyroid hormone
IL-11	Interleukin-11
TGF-β	Transforming growth factor-beta
RA	Rheumatoid arthritis
HSPCs	Hematopoietic stem/progenitor cells
NHO	Neurogenic heterotopic
OIM	Osteoimmunomodulatory
MSCs	Mesenchymal stem cells

## References

[1] Okamoto K. (2024). Crosstalk between bone and the immune system. J. Bone Min. Metab..

[2] Zhou F., Zhang G., Wu Y., Xiong Y. (2022). Inflammasome Complexes: Crucial mediators in osteoimmunology and bone diseases. Int. Immunopharmacol..

[3] Wawrzyniak A., Balawender K. (2022). Structural and Metabolic Changes in Bone. Animals.

[4] Lucas D. (2021). Structural organization of the bone marrow and its role in hematopoiesis. Curr. Opin. Hematol..

[5] Pedrero S.G., Llamas-Sillero P., Serrano-López J. (2021). A Multidisciplinary Journey towards Bone Tissue Engineering. Materials.

[6] Boyce B.F. (2013). Advances in osteoclast biology reveal potential new drug targets and new roles for osteoclasts. J. Bone Min. Res..

[7] Yang N., Liu Y. (2021). The Role of the Immune Microenvironment in Bone Regeneration. Int. J. Med. Sci..

[8] Arun M., Rajasingh S., Madasamy P., Rajasingh J. (2025). Immunomodulatory and Regenerative Functions of MSC-Derived Exosomes in Bone Repair. Bioengineering.

[9] Takayanagi H. (2007). Osteoimmunology: Shared mechanisms and crosstalk between the immune and bone systems. Nat. Rev. Immunol..

[10] Okamoto K., Nakashima T., Shinohara M., Negishi-Koga T., Komatsu N., Terashima A., Sawa S., Nitta T., Takayanagi H. (2017). Osteoimmunology: The conceptual framework unifying the immune and skeletal systems. Physiol. Rev..

[11] Mensah K.A., Li J., Schwarz E.M. (2009). The emerging field of osteoimmunology. Immunol. Res..

[12] Liu L., Chen H., Zhao X., Han Q., Xu Y., Liu Y., Zhang A., Li Y., Zhang W., Chen B. (2024). Advances in the application and research of biomaterials in promoting bone repair and regeneration through immune modulation. Mater. Today Bio.

[13] Zhang W., Zeng X., Deng X., Yang F., Ma X., Gao W. (2025). Smart biomaterials: As active immune modulators to shape pro-regenerative microenvironments. Front. Cell Dev. Biol..

[14] Calabrese G., Petralia S., Fabbi C., Forte S., Franco D., Guglielmino S., Esposito E., Cuzzocrea S., Traina F., Conoci S. (2020). Au, Pd and maghemite nanofunctionalized hydroxyapatite scaffolds for bone regeneration. Regen. Biomater..

[15] Mauro N., Calabrese G., Sciortino A., Rizzo M.G., Messina F., Giammona G., Cavallaro G. (2024). Microporous Fluorescent Poly(D,L-lactide) Acid-Carbon Nanodot Scaffolds for Bone Tissue Engineering Applications. Materials.

[16] Munaò S., D’Amora U., Bauso L.V., Ronca A., Manini P., Pezzella A., Raucci M.G., Ambrosio L., Calabrese G. (2025). In Vitro Osteogenic Stimulation of Human Adipose-Derived MSCs on Biofunctional 3D-Printed Scaffolds. Biomedicines.

[17] Mi B., Xiong Y., Zha K., Cao F., Zhou W., Abbaszadeh S., Ouyang L., Liao Y., Hu W., Dai G. (2023). Immune homeostasis modulation by hydrogel-guided delivery systems: A tool for accelerated bone regeneration. Biomater. Sci..

[18] Downey P.A., Siegel M.I. (2006). Bone biology and the clinical implications for osteoporosis. Phys. Ther..

[19] Ben Letaifa R., Klaylat T., Tarchala M., Gao C., Schneider P., Rosenzweig D.H., Martineau P.A., Gawri R. (2024). Osteoimunology: An Overview of the Interplay of the Immune System and the Bone Tissue in Fracture Healing. Surgeries.

[20] Robling A.G., Castillo A.B., Turner C.H. (2006). Biomechanical and molecular regulation of bone remodeling. Annu. Rev. Biomed. Eng..

[21] Florencio-Silva R., Sasso G.R., Sasso-Cerri E., Simões M.J., Cerri P.S. (2015). Biology of Bone Tissue: Structure, Function, and Factors That Influence Bone Cells. BioMed Res. Int..

[22] Lorenzo J., Horowitz M., Choi Y. (2008). Osteoimmunology: Interactions of the bone and immune system. Endocr. Rev..

[23] Mori G., D’Amelio P., Faccio R., Brunetti G. (2013). The Interplay between the bone and the immune system. Clin. Dev. Immunol..

[24] Arron J.R., Choi Y. (2000). Bone versus Immune System. Nature.

[25] Zou Y., Xie Q., Lin J., Dong H., Zhuang X., Xian R., Liang Y., Li S. (2025). Immunomodulatory Effects and Mechanisms of Two-Dimensional Black Phosphorus on Macrophage Polarization and Bone Regeneration. Int. J. Nanom..

[26] Wang Y.-H., Zhao C.-Z., Wang R.-Y., Du Q.-X., Liu J.-Y., Pan J. (2022). The crosstalk between macrophages and bone marrow mesenchymal stem cells in bone healing. Stem Cell Res. Ther..

[27] Lin X., Patil S., Gao Y.G., Qian A. (2020). The Bone Extracellular Matrix in Bone Formation and Regeneration. Front. Pharmacol..

[28] Šromová V., Sobola D., Kaspar P. (2023). A Brief Review of Bone Cell Function and Importance. Cells.

[29] Mizoguchi T., Ono N. (2021). The diverse origin of bone-forming osteoblasts. J. Bone Min. Res..

[30] Arvidson K., Abdallah B.M., Applegate L.A., Baldini N., Cenni E., Gomez-Barrena E., Granchi D., Kassem M., Konttinen Y.T., Mustafa K. (2011). Bone regeneration and stem cells. J. Cell. Mol. Med..

[31] He T., Qin L., Chen S., Huo S., Li J., Zhang F., Yi W., Mei Y., Xiao G. (2025). Bone-derived factors mediate crosstalk between skeletal and extra-skeletal organs. Bone Res..

[32] Iaquinta M.R., Montesi M., Mazzoni E. (2024). Advances in Bone Biology. Int. J. Mol. Sci..

[33] Borciani G., Montalbano G., Baldini N., Cerqueni G., Vitale-Brovarone C., Ciapetti G. (2020). Co-culture systems of osteoblasts and osteoclasts: Simulating in vitro bone remodeling in regenerative approaches. Acta Biomater..

[34] Kular J., Tickner J., Chim S.M., Xu J. (2012). An overview of the regulation of bone remodelling at the cellular level. Clin. Biochem..

[35] Raggatt L.J., Partridge N.C. (2010). Cellular and Molecular Mechanisms of Bone Remodeling. J. Biol. Chem..

[36] Katsimbri P. (2017). The Biology of Normal Bone Remodelling. Eur. J. Cancer Care.

[37] Bolamperti S., Villa I., Rubinacci A. (2022). Bone remodeling: An operational process ensuring survival and bone mechanical competence. Bone Res..

[38] Kenkre J.S., Bassett J. (2018). The Bone Remodelling Cycle. Ann. Clin. Biochem..

[39] Sims N.A., Martin T.J. (2014). Coupling the activities of bone formation and resorption: A multitude of signals within the basic multicellular unit. BoneKEy Rep..

[40] Singh A., Mehdi A.A., Srivastava R.N., Verma N.S. (2012). Immunoregulation of bone remodelling. Int. J. Crit. Illn. Inj. Sci..

[41] Xu J., Yu L., Liu F., Wan L., Deng Z. (2023). The effect of cytokines on osteoblasts and osteoclasts in bone remodeling in osteoporosis: A review. Front. Immunol..

[42] Epsley S., Tadros S., Farid A., Kargilis D., Mehta S., Rajapakse C.S. (2021). The Effect of Inflammation on Bone. Front. Physiol..

[43] Zhao B., Ivashkiv L.B. (2011). Negative regulation of osteoclastogenesis and bone resorption by cytokines and transcriptional repressors. Arthritis Res. Ther..

[44] Xue N., Ding X., Huang R., Jiang R., Huang H., Pan X., Min W., Chen J., Duan J.A., Liu P. (2022). Bone Tissue Engineering in the Treatment of Bone Defects. Pharmaceuticals.

[45] Rastogi S., Verma R., Gouru S.A., Venkatesan K., Pandian P.M., Khan M.I., Deka T., Kumar P. (2025). Emerging Technologies in Bone Tissue Engineering: A Review. J. Bionic Eng..

[46] Bauso L.V., La Fauci V., Longo C., Calabrese G. (2024). Bone Tissue Engineering and Nanotechnology: A Promising Combination for Bone Regeneration. Biology.

[47] Rizzo M.G., Palermo N., Alibrandi P., Sciuto E.L., Del Gaudio C., Filardi V., Fazio B., Caccamo A., Oddo S., Calabrese G. (2023). Physiologic Response Evaluation of Human Foetal Osteoblast Cells within Engineered 3D-Printed Polylactic Acid Scaffolds. Biology.

[48] Calabrese G., Franco D., Petralia S., Monforte F., Condorelli G.G., Squarzoni S., Traina F., Conoci S. (2021). Dual-Functional Nano-Functionalized Titanium Scaffolds to Inhibit Bacterial Growth and Enhance Osteointegration. Nanomaterials.

[49] Rahaman J., Mukherjee D. (2025). Osteoimmunomodulatory biomaterials: Engineering strategies, current progress, and future perspectives for bone regeneration. Appl. Mater. Today.

[50] Chaplin D.D. (2010). Overview of the immune response. J. Allergy Clin. Immunol..

[51] Shevyrev D., Tereshchenko V., Berezina T.N., Rybtsov S. (2023). Hematopoietic Stem Cells and the Immune System in Development and Aging. Int. J. Mol. Sci..

[52] Ruffinatto L., Groult Y., Iacono J., Sarrazin S., de Laval B. (2024). Hematopoietic stem cell a reservoir of innate immune memory. Front. Immunol..

[53] Wicherska-Pawłowska K., Wróbel T., Rybka J. (2021). Toll-Like Receptors (TLRs), NOD-Like Receptors (NLRs), and RIG-I-Like Receptors (RLRs) in Innate Immunity. TLRs, NLRs, and RLRs Ligands as Immunotherapeutic Agents for Hematopoietic Diseases. Int. J. Mol. Sci..

[54] Yin L., Sun C., Zhang J., Li Y., Wang Y., Bai L., Lei Z. (2025). Critical signaling pathways in osteoclast differentiation and bone resorption: Mechanisms and therapeutic implications for periprosthetic osteolysis. Front. Cell Dev. Biol..

[55] Ben-awadh A.N., Delgado-Calle J., Tu X., Kuhlenschmidt K., Allen M.R., Plotkin L.I., Bellido T. (2014). Parathyroid hormone receptor signaling induces bone resorption in the adult skeleton by directly regulating the RANKL gene in osteocytes. Endocrinology.

[56] Feng X., Lv C., Wang F., Gan K., Zhang M., Tan W. (2013). Modulatory effect of 1,25-dihydroxyvitamin D 3 on IL1 β -induced RANKL, OPG, TNF α, and IL-6 expression in human rheumatoid synoviocyte MH7A. Clin. Dev. Immunol..

[57] Marahleh A., Kitaura H., Ohori F., Kishikawa A., Ogawa S., Shen W.R., Qi J., Noguchi T., Nara Y., Mizoguchi I. (2019). TNF-α Directly Enhances Osteocyte RANKL Expression and Promotes Osteoclast Formation. Front. Immunol..

[58] Jura-Półtorak A., Szeremeta A., Olczyk K., Zoń-Giebel A., Komosińska-Vassev K. (2021). Bone Metabolism and RANKL/OPG Ratio in Rheumatoid Arthritis Women Treated with TNF-α Inhibitors. J. Clin. Med..

[59] Kwan T.S., Padrines M., The′oleyre S., Heymann D., Fortun Y. (2004). IL-6, RANKL, TNF-alpha/IL-1: Interrelations in bone resorption pathophysiology. Cytokine Growth Factor. Rev..

[60] Fukawa Y., Kayamori K., Tsuchiya M., Ikeda T. (2023). IL-1 Generated by Oral Squamous Cell Carcinoma Stimulates Tumor-Induced and RANKL-Induced Osteoclastogenesis: A Possible Mechanism of Bone Resorption Induced by the Infiltration of Oral Squamous Cell Carcinoma. Int. J. Mol. Sci..

[61] Feng W., Yang P., Liu H., Zhang F., Li M. (2022). IL-6 promotes low concentration of RANKL-induced osteoclastic differentiation by mouse BMMs through trans-signaling pathway. J. Mol. Histol..

[62] Hill P.A., Tumber A., Papaioannou S., Meikle M.C. (1998). The Cellular Actions of Interleukin-11 on Bone Resorption in Vitro. Endocrinology.

[63] Han Y., Gao H., Gan X., Liu J., Bao C., He C. (2024). Roles of IL-11 in the regulation of bone metabolism. Front. Endocrinol..

[64] Pacifici R. (2016). The Role of IL-17 and TH17 Cells in the Bone Catabolic Activity of PTH. Front. Immunol..

[65] Li J.Y., Yu M., Tyagi A.M., Vaccaro C., Hsu E., Adams J., Bellido T., Weitzmann M.N., Pacifici R. (2019). IL-17 Receptor Signaling in Osteoblasts/Osteocytes Mediates PTH-Induced Bone Loss and Enhances Osteocytic RANKL Production. J. Bone Min. Res..

[66] Zhao J.J., Wu Z.F., Yu Y.H., Wang L., Cheng L. (2018). Effects of interleukin-7/interleukin-7 receptor on RANKL-mediated osteoclast differentiation and ovariectomy-induced bone loss by regulating c-Fos/c-Jun pathway. J. Cell. Physiol..

[67] Choudhary S., Blackwell K., Voznesensky O., Deb Roy A., Pilbeam C. (2013). Prostaglandin E2 acts via bone marrow macrophages to block PTH-stimulated osteoblast differentiation in vitro. Bone.

[68] Mano M., Arakawa T., Mano H., Nakagawa M., Kaneda T., Kaneko H., Yamada T., Miyata K., Kiyomura H., Kumegawa M. (2000). Prostaglandin E2 directly inhibits bone resorbing activity of isolated mature osteoclasts mainly through the EP4 receptor. Calcif. Tissue Int..

[69] Mun S.H., Park P.S.U., Park-Min K.H. (2020). The M-CSF receptor in osteoclasts and beyond. Exp. Mol. Med..

[70] Ibáñez L., Nácher-Juan J., Terencio M.C., Ferrándiz M.L., Alcaraz M.J. (2022). Osteostatin Inhibits M-CSF+RANKL-Induced Human Osteoclast Differentiation by Modulating NFATc1. Int. J. Mol. Sci..

[71] Stein N.C., Kreutzmann C., Zimmermann S.-P., Niebergall U., Hellmeyer L., Goettsch C., Schoppet M., Hofbauer L.C. (2008). Interleukin-4 and interleukin-13 stimulate the osteoclast inhibitor osteoprotegerin by human endothelial cells through the STAT6 pathway. J. Bone Min. Res..

[72] Mirosavljevic D., Quinn J.M., Elliott J., Horwood N.J., Martin T.J., Gillespie M.T. (2003). T-cells mediate an inhibitory effect of interleukin-4 on osteoclastogenesis. J. Bone Min. Res..

[73] Takayanagi H., Kim S., Taniguchi T. (2002). Signaling crosstalk between RANKL and interferons in osteoclast differentiation. Arthritis Res..

[74] Ji J.D., Park-Min K.H., Shen Z., Fajardo R.J., Goldring S.R., McHugh K.P., Ivashkiv L.B. (2009). Inhibition of RANK expression and osteoclastogenesis by TLRs and IFN-gamma in human osteoclast precursors. J. Immunol..

[75] Evans K.E., Fox S.W. (2007). Interleukin-10 inhibits osteoclastogenesis by reducing NFATc1 expression and preventing its translocation to the nucleus. BMC Cell Biol..

[76] Gao Y., Grassi F., Ryan M.R., Terauchi M., Page K., Yang X., Weitzmann M.N., Pacifici R. (2007). IFN-gamma stimulates osteoclast formation and bone loss in vivo via antigen-driven T cell activation. J. Clin. Investig..

[77] Kitaura H., Marahleh A., Ohori F., Noguchi T., Shen W.-R., Qi J., Nara Y., Pramusita A., Kinjo R., Mizoguchi I. (2020). Osteocyte-Related Cytokines Regulate Osteoclast Formation and Bone Resorption. Int. J. Mol. Sci..

[78] Dalle Carbonare L., Cominacini M., Trabetti E., Bombieri C., Pessoa J., Romanelli M.G., Valenti M.T. (2025). The bone microenvironment: New insights into the role of stem cells and cell communication in bone regeneration. Stem Cell Res. Ther..

[79] Zhu X., Fan Y., Wang W., Wu R., Zhang C., Yang Z. (2025). Unraveling the causal role of TGF-βRII in osteoporosis and the potential of its associated differential genes as novel targets. Eur. J. Med. Res..

[80] Tokunaga T. (2020). TGFβ1 Regulates Human RANKL-Induced Osteoclastogenesis via Sup-pression of NFATc1 Expression. Int. J. Mol. Sci..

[81] Boyce B.F., Xing L. (2008). Functions of RANKL/RANK/OPG in bone modeling and remodeling. Arch. Biochem. Biophys..

[82] Infante M., Fabi A., Cognetti F., Gorini S., Caprio M., Fabbri A. (2019). RANKL/RANK/OPG system beyond bone remodeling: Involvement in breast cancer and clinical perspectives. J. Exp. Clin. Cancer Res..

[83] Sun Y., Li J., Xie X., Gu F., Sui Z., Zhang K., Yu T. (2021). Macrophage-Osteoclast Associations: Origin, Polarization, and Subgroups. Front. Immunol..

[84] Yang X., Pande S., Scott C., Friesel R. (2019). Macrophage colony-stimulating factor pretreatment of bone marrow progenitor cells regulates osteoclast differentiation based upon the stage of myeloid development. J. Cell. Biochem..

[85] Wu Z., Li W., Jiang K., Lin Z., Qian C., Wu M., Xia Y., Li N., Zhang H., Xiao H. (2024). Regulation of bone homeostasis: Signaling pathways and therapeutic targets. MedComm.

[86] Yahara Y., Nguyen T., Ishikawa K., Kamei K., Alman B.A. (2022). The origins and roles of osteoclasts in bone development, homeostasis and repair. Development.

[87] Yao Y., Cai X., Ren F., Ye Y., Wang F., Zheng C., Qian Y., Zhang M. (2021). The Macrophage-Osteoclast Axis in Osteoimmunity and Osteo-Related Diseases. Front. Immunol..

[88] Grčević D., Sanjay A., Lorenzo J. (2023). Interactions of B-lymphocytes and bone cells in health and disease. Bone.

[89] Gu Q., Yang H., Shi Q. (2017). Macrophages and bone inflammation. J. Orthop. Translat..

[90] Li H., Hong S., Qian J., Zheng Y., Yang J., Yi Q. (2010). Cross talk between the bone and immune systems: Osteoclasts function as antigen-presenting cells and activate CD4+ and CD8+ T cells. Blood.

[91] Jung S.M., Kim K.W., Yang C.W., Park S.H., Ju J.H. (2014). Cytokine-mediated bone destruction in rheumatoid arthritis. J. Immunol. Res..

[92] Xu H., Wang W., Liu X., Huang W., Zhu C., Xu Y., Yang H., Bai J., Geng D. (2023). Targeting strategies for bone diseases: Signaling pathways and clinical studies. Signal Transduct. Target. Ther..

[93] Guder C., Gravius S., Burger C., Wirtz D.C., Schildberg F.A. (2020). Osteoimmunology: A Current Update of the Interplay Between Bone and the Immune System. Front. Immunol..

[94] Li Y., Terauchi M., Vikulina T., Roser-Page S., Weitzmann M.N. (2014). B Cell Production of Both OPG and RANKL is Significantly Increased in Aged Mice. Open Bone J..

[95] Sözen T., Özışık L., Başaran N.Ç. (2017). An overview and management of osteoporosis. Eur. J. Rheumatol..

[96] Dimai H.P., Fahrleitner-Pammer A. (2022). Osteoporosis and Fragility Fractures: Currently available pharmacological options and future directions. Best. Pract. Res. Clin. Rheumatol..

[97] Weitzmann M.N., Ofotokun I. (2016). Physiological and pathophysiological bone turnover—Role of the immune system. Nat. Rev. Endocrinol..

[98] Saxena Y., Routh S., Mukhopadhaya A. (2021). Immunoporosis: Role of Innate Immune Cells in Osteoporosis. Front. Immunol..

[99] Komatsu N., Takayanagi H. (2012). Autoimmune arthritis: The interface between the immune system and joints. Adv. Immunol..

[100] Hajishengallis G., Chavakis T., Lambris J.D. (2020). Current understanding of periodontal disease pathogenesis and targets for host-modulation therapy. Periodontology 2000.

[101] Satcher R.L., Zhang X.H. (2022). Evolving cancer-niche interactions and therapeutic targets during bone metastasis. Nat. Rev. Cancer.

[102] Ferraro F., Lymperi S., Méndez-Ferrer S., Saez B., Spencer J.A., Yeap B.Y., Masselli E., Graiani G., Prezioso L., Rizzini E.L. (2011). Diabetes impairs hematopoietic stem cell mobilization by altering niche function. Sci. Transl. Med..

[103] Wong K.R., Mychasiuk R., O’Brien T.J., Shultz S.R., McDonald S.J., Brady R.D. (2020). Neurological heterotopic ossification: Novel mechanisms, prognostic biomarkers and prophylactic therapies. Bone Res..

[104] Weitzmann M.N. (2017). Bone and the Immune System. Toxicol. Pathol..

[105] Li J., Qu Y., Chu B., Wu T., Pan M., Mo D., Li L., Ming Y., Yang Y., Wang M. (2025). Research Progress on Biomaterials with Immunomodulatory Effects in Bone Regeneration. Adv. Sci..

[106] Ouyang L., Cao J., Dai Q., Qiu D. (2021). New insight of immuno-engineering in osteoimmunomodulation for bone regeneration. Regen. Ther..

[107] Carrascal-Hernández D.C., Martínez-Cano J.P., Rodríguez Macías J.D., Grande-Tovar C.D. (2025). Evolution in Bone Tissue Regeneration: From Grafts to Innovative Biomaterials. Int. J. Mol. Sci..

[108] Tupe A., Patole V., Ingavle G., Kavitkar G., Mishra Tiwari R., Kapare H., Baheti R., Jadhav P. (2024). Recent Advances in Biomaterial-Based Scaffolds for Guided Bone Tissue Engineering: Challenges and Future Directions. Polym. Adv. Technol..

[109] Abaricia J.O., Farzad N., Heath T.J., Simmons J., Morandini L., Olivares-Navarrete R. (2021). Control of innate immune response by biomaterial surface topography, energy, and stiffness. Acta Biomater..

[110] Sheikh Z., Brooks P.J., Barzilay O., Fine N., Glogauer M. (2015). Macrophages, Foreign Body Giant Cells and Their Response to Implantable Biomaterials. Materials.

[111] Negrescu A.M., Cimpean A. (2021). The State of the Art and Prospects for Osteoimmunomodulatory Biomaterials. Materials.

[112] Chen Z., Xing F., Zhou Y., Yu P., Xu J., Luo R., Zhou C., Xiang Z., Rommens P.M., Liu M. (2023). Integrated osteoimmunomodulatory strategies based on designing scaffold surface properties in bone regeneration. J. Mater. Chem. B.

[113] He J., Chen G., Liu M., Xu Z., Chen H., Yang L., Lv Y. (2020). Scaffold strategies for modulating immune microenvironment during bone regeneration. Mater. Sci. Eng. C. Mater. Biol..

[114] Xiong Y., Xiong Y. (2022). Applications of bone regeneration hydrogels in the treatment of bone defects: A review. J. Mater. Sci..

[115] Zheng Y., Ke Z., Hu G., Tong S. (2024). Hydrogel promotes bone regeneration through various mechanisms: A review. Biomed. Tech..

[116] Zhang R., Tan S.F., Wang Y., Wu J., Zhang C. (2025). From Macrophage Polarization to Clinical Translation: Immunomodulatory Hydrogels for Infection-Associated Bone Regeneration. Front. Cell Dev. Biol..

[117] Biglari N., Razzaghi M., Afkham Y., Azimi G., Gross J.D., Samadi A. (2025). Advanced biomaterials in immune modulation: The future of regenerative therapies. Int. J. Pharm..

